# Camel Milk Targeting Insulin Receptor—Toward Understanding the Antidiabetic Effects of Camel Milk

**DOI:** 10.3389/fnut.2021.819278

**Published:** 2022-02-09

**Authors:** Irfa Anwar, Farheen Badrealam Khan, Sajid Maqsood, Mohammed Akli Ayoub

**Affiliations:** ^1^Department of Biology, College of Science, The United Arab Emirates University, Al Ain, United Arab Emirates; ^2^Department of Food Science, College of Agriculture and Veterinary Medicine, The United Arab Emirates University, Al Ain, United Arab Emirates; ^3^Zayed Center for Health Sciences, The United Arab Emirates University, Al Ain, United Arab Emirates

**Keywords:** camel milk, whey proteins, insulin receptor, diabetes, bioactive peptides, BRET, lactoferrin

## Abstract

Camel milk (CM) is known for its beneficial virtues in the human diet and health. This includes its antidiabetic properties demonstrated in many *in vitro* and *in vivo* studies. Nevertheless, the scientific rationale behind the molecular and cellular basis of such beneficial effects and the exact antidiabetic agent(s)/mechanism(s) are still elusive. In this review, we focused on the recent advances supporting the targeting of insulin receptor (IR) by CM components. Indeed, our recent work reported that CM proteins and derived peptides pharmacologically target IR *in vitro* leading to its activation and potentiation of insulin-mediated responses. The review describes the experimental approaches used to investigate the effects of CM on IR *in vitro* based on the fractionation of CM whey proteins to purify functional proteins and their hydrolysis by gastric proteases to generate bioactive peptides. In addition, we illustrated our cellular and molecular model consisting of studying the functional activity of CM fractions on IR and its downstream signaling pathways in the hepatocarcinoma (HepG2) and the human embryonic kidney (HEK293) cells using the bioluminescence resonance energy transfer (BRET), phosphorylation, and glucose uptake assays. Overall, our work demonstrated for the first time that CM lactoferrin and CM-derived bioactive peptides positively modulate IR and its related signaling pathways in HepG2 and HEK293 cells. As a conclusion, the pharmacological targeting of IR by CM sheds more light on the antidiabetic properties of CM by providing its molecular basis that may constitute a solid rationale for the development of new generation of antidiabetic tools from CM-derived proteins and peptides and the utilization of CM in the management of diabetes. The sequencing and the synthesis of the potent bioactive CM peptides may open promising perspectives for their application as antidiabetic agents.

## Introduction

**Figure 1:**
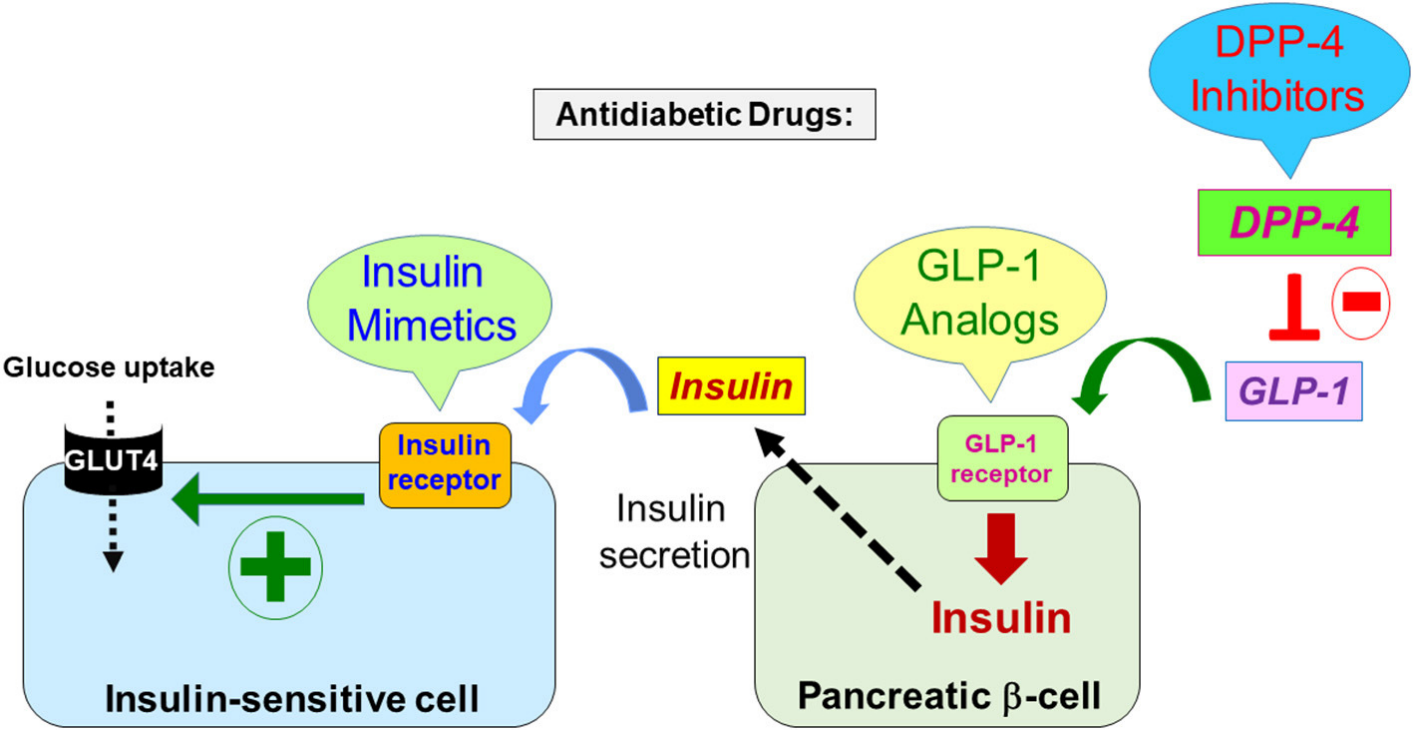
Representative figure depicting some of the current antidiabetic strategies along with their molecular targets. At the molecular level, the major antidiabetic drugs aim either to increase the production and release of insulin by the pancreatic beta cells or to potentiate its biological activity in the major targets. Thus, the therapeutic molecules may be either insulin mimetics targeting and activating the insulin receptor in the insulin-sensitive cells that eventually leads to glucose uptake *via* the glucose transporter GLUT4; or GLP-1 receptor analogs that increase insulin synthesis and its secretion in the pancreatic beta-cell; or the inhibitors of the enzyme DPP-4 that result in the increase of GLP-1 levels controlling insulin synthesis and secretion *via* binding and activation of its receptor (GLP-1 receptor).

**Figure 2:**
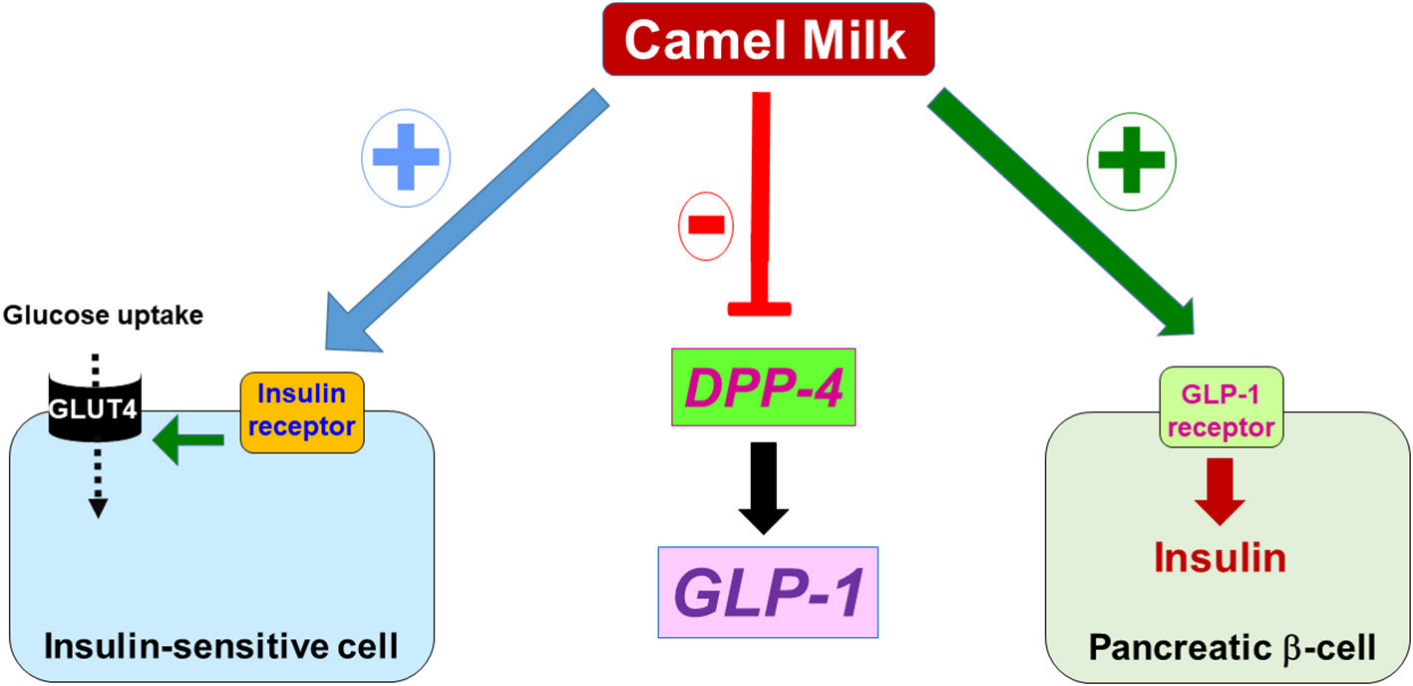
Possible molecular targets of CM in the context of diabetes. At the molecular level, the antidiabetic effects of CM components might seemingly be mediated through targeting and/or activating (+) the insulin receptor in the insulin-sensitive cells that leads to glucose uptake *via* the glucose transporter GLUT4; or activating GLP-1 receptor (+) in the pancreatic beta cell that increases insulin synthesis and secretion, or by inhibiting the enzyme DPP-4 which results in the increase of GLP-1 levels controlling insulin synthesis and secretion *via* binding and activation of its receptor (GLP-1 receptor).

Camel milk (CM) has been reported to have many interesting dietetic, medicinal, and social benefits. This was shown to be linked to its chemical and molecular composition and properties as documented by many analytical studies (1–5). Thus, it is now accepted that CM has beneficial effects in human health with potential applications in various diseases and disorders including diabetes, cancer, inflammation, hypertension, infection (6–10). The antidiabetic properties of CM constitute the most studied aspect of CM using *in vitro* approaches as well as *in vivo* studies on animal models and diabetic patients (10–14). In these studies, various aspects related to diabetes such as glycemia, lipid profile, insulin secretion and resistance were studied and were found to be significantly improved by CM. In humans, the beneficial effects of CM in diabetes and its related disorders were even examined in many randomized clinical studies (9, 14–20). Mirmiran et al., have provided a comprehensive compilation of various animal and clinical studies highlighting the role of CM against diabetic complications (19). Overall, these studies reported that feeding the diabetic patients with CM significantly improved various important biochemical and clinical parameters in diabetic pathology. In addition, these clinical trials reported that the dose of insulin required to the normalize glucose levels in blood was reduced in diabetic patients who received CM. Zheng et al. recently reported an elegant clinical study on 14 type-2 diabetic patients supplemented with CM powder and showing CM decreased fasting blood glucose, serum cholesterol (14). By contrast, CM increased in serum content of the glucagon-like peptide 1 (GLP-1), known as a key regulator of insulin secretion by the pancreas (14). Moreover, a comprehensive and a comparative study on one tribe of camel breeders in India who frequently consume CM showed a lower incidence risk of diabetes compared to other communities; plausibly due to consumption of CM (21). This study along with others advocates for the antidiabetic potential of CM (21–23). However, the scientific rationale behind such antidiabetic properties of CM is still elusive with regard to two key aspects. The first aspect refers to the fact that the potential antidiabetic agent(s) contained in CM have yet to be identified and characterized. Since insulin constitutes the pivotal hormone regulating glucose homeostasis, some studies speculated about the existence of insulin and/or insulin-like molecules in CM at different concentrations over the breeds, the lactation stages, and storage conditions, that may explain its antidiabetic effects (24–27). It has been reported that CM contains 3 times more insulin-like proteins than cow milk (28, 29). However, a recent study reported that the digestive enzymes completely reduced the intact CM insulin as well as its activity after 30 minutes suggesting that insulin should not reach the blood circulation since it would be fully degraded by the digestive tract proteases and thereby it may not be involved in the antidiabetic action of CM (24, 30, 31). Thus, the exact insulin levels in CM and its putative implication in the antidiabetic effects of CM is still a matter of debate and further studies are required to address this important aspect. Recent analytical studies on CM fractionation and processing suggest the existence of bioactive molecules (proteins/peptides) toward the key pathways involved in glucose homeostasis including insulin secretion by the pancreatic beta-cells as well as insulin action at the level of the major target tissues (liver, skeletal muscles, adipocytes) (32–39). Moreover, other studies reported a link between diabetes and some CM components and proteins such as lactoferrin (40–44). The second aspect is related to the exact molecular and cellular mechanisms engaged by CM components that are not fully elucidated. From this point of view, several studies attempted to investigate the effect of CM at the cellular and molecular levels using various *in vitro* and/or *in vivo* models. Obviously, in the context of diabetes and antidiabetic approaches/drugs; different modes of action are possible at the molecular and the cellular levels that are known to be involved in glucose homeostasis and metabolism (Figure 1). This includes the therapeutic molecules that target the well-known key molecular components and mediators controlling pathways related to insulin and its receptor (IR) and GLP-1 and its receptor (GLP-1R) and the dipeptidyl peptidase 4 (DPP-4) enzyme, both known to differentially control insulin synthesis and secretion through activation or inhibition, respectively (Figure 1). Accordingly, the antidiabetic properties of CM may be mediated by active molecules that directly or indirectly target and modulate insulin secretion and/or insulin action pathways (Figure 2). As for insulin secretion, many *in vitro* and *in vivo* studies have indeed reported that CM increases insulin secretion (15, 22, 45, 46). This may involve an effect on the pancreatic beta-cells and their functions such as the stimulation of insulin synthesis and release and/or their protection from damage and apoptotic pathways. Indeed, CM may have multiple key molecular targets including the activation of GLP-1 and its receptor and/or the inhibition of DPP-4, which is known to downregulate GLP-1 (Figure 2). Previous studies have explicitly demonstrated that CM-derived protein, hydrolysates and peptides can inhibit DPP-4 and modulate GLP-1 levels (37, 38, 45, 47). On the other hand, studies reported the protective actions of CM on the pancreatic beta-cells against damage caused by inflammation and redox reactions involving immunomodulatory, anti-inflammatory, anti-oxidative pathways (45, 46, 48, 49). Further, regarding the putative effect of CM on insulin action and pathways, as stated above insulin is the key hormone controlling glucose uptake and metabolism in the major insulin-sensitive organs and tissues. Therefore, it is legitimate to speculate about the possible targeting of insulin and its receptor and its molecular pathways by CM (Figure 2). This may constitute a valid possibility based on *in vivo* studies reporting that CM did not affect the circulating insulin levels, but rather increased its response resulting in lower insulin doses required to control glycemia in diabetic rats (22, 50). Moreover, our recent *in vitro* work revealed the targeting and the positive modulation of IR and its intracellular signaling pathway by CM proteins and peptides (32, 33). This constitutes the main topic of our review as discussed and described below.

## *In Vitro* Experimental Approach

**Figure 3:**
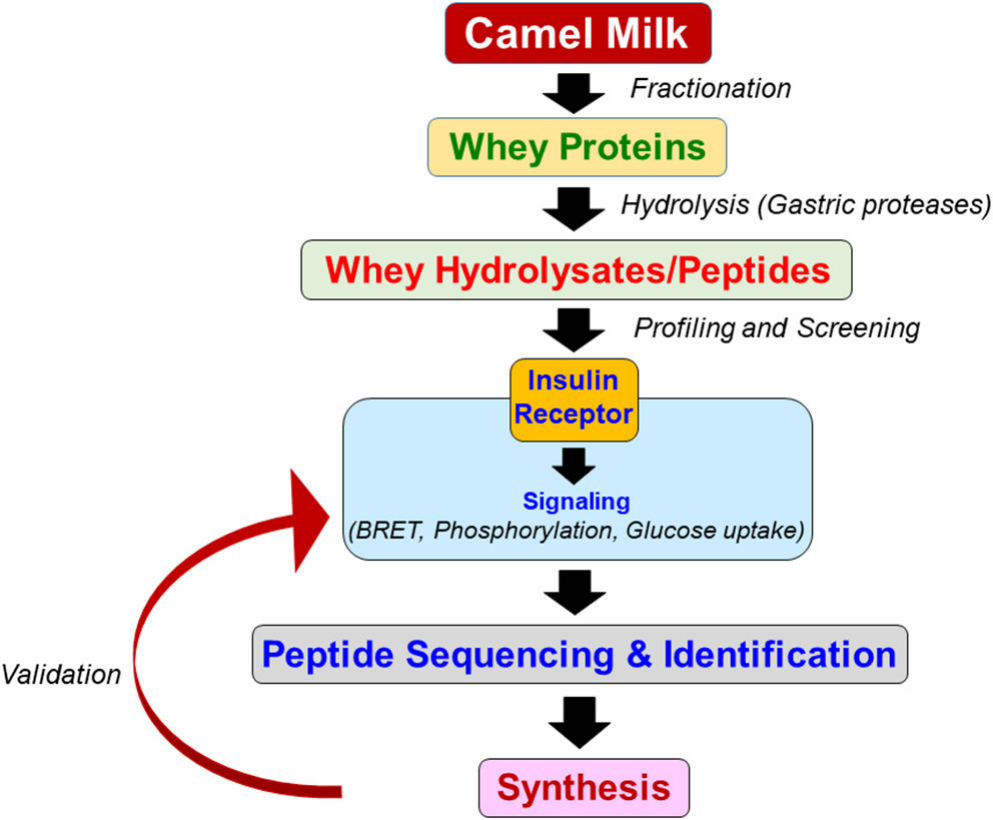
Flowchart of the experimental approach used for CM fractionation and profiling. The raw whey proteins are firstly separated and extracted from freshly collected CM. Thereafter, the proteins were subjected to hydrolysis using the major gastric endopeptidases resulting in various hydrolysates obtained from different experimental protocols. The CM whey hydrolysates and derived peptides are then screened and profiled for their bioactivity and functional action on IR using cell lines and employing different assays including BRET, phosphorylation assay, and glucose uptake. The most active and potent hydrolysates and peptides are sequenced for peptide content and identification. Finally, the most relevant peptides are synthesized and validated again on IR activity using cellular model.

In our work, we investigated the effect of CM on IR function *in vitro* using a cellular system. For this, the whole or fractionated forms of CM as well as intact purified CM proteins were used (32, 33, 51). Indeed, the focus was on the CM proteins based on the hypothesis that the protein fraction may be a prominent source for bioactive protein/peptides responsible for the antidiabetic action of CM as previously reported (34, 36–39, 47). Moreover, the gastric digestion and processing of CM upon its consumption constitutes a pertinent question and aspect to be considered to gauge the antidiabetic action of CM in real manner as reported *in vivo*. Indeed, CM components including proteins would be subjected to gastric proteolytic degradation releasing peptide and/or amino acid molecules in the gut, which would be followed by their intestinal absorption, and eventually release into the circulation to exert their biological and pharmacological action in the different target tissues. With this line, the bioactive antidiabetic agent(s) of CM may result from such a proteolysis process in the gut being consistent with the beneficial effects of CM in various *in vivo* diabetic models. Indeed, the earlier notion of the presence of insulin/insulin-like peptides in intact form and their resistance to gastrointestinal digestion has been recently revised by independent studies that explicitly highlighted that these insulin/insulin-like peptides does not resist to the proteolytic digestion (24, 30, 31). This constitutes the rationale behind generating CM hydrolysates and their profiling and screening for the potential targeting of IR (Figure 3). However, none can exclude the possibility that some CM proteins resist to proteolysis and stay as intact bioactive proteins triggering the antidiabetic effects of CM. The possible encapsulation of CM proteins in some sort of lipid based nano-vesicles in the digestive system was also envisaged (30). Accordingly, we considered lactoferrin (LF) as one known bioactive milk protein (52–55) that we successfully purified from CM and profiled as functionally active on IR (32). LF as intact protein is known to have therapeutic potential in various human diseases including diabetes (42, 56–58). In addition, LF was reported to resist to gastric digestion and stay intact for its interaction with its specific receptor in the small intestine (58). Collectively, it is reasonable to study both intact CM proteins as well as hydrolysates and accordingly, both intact CM whey proteins and their derived hydrolysates are isolated, profiled and screened for their bioactivity on IR function *in vitro* as shown in Figure 3. Furthermore, the CM hydrolysates are sequenced and peptide identification is carried out for further validation on IR activity (Figure 3).

## *In Vitro* Model for Investigating IR Function

**Figure 4:**
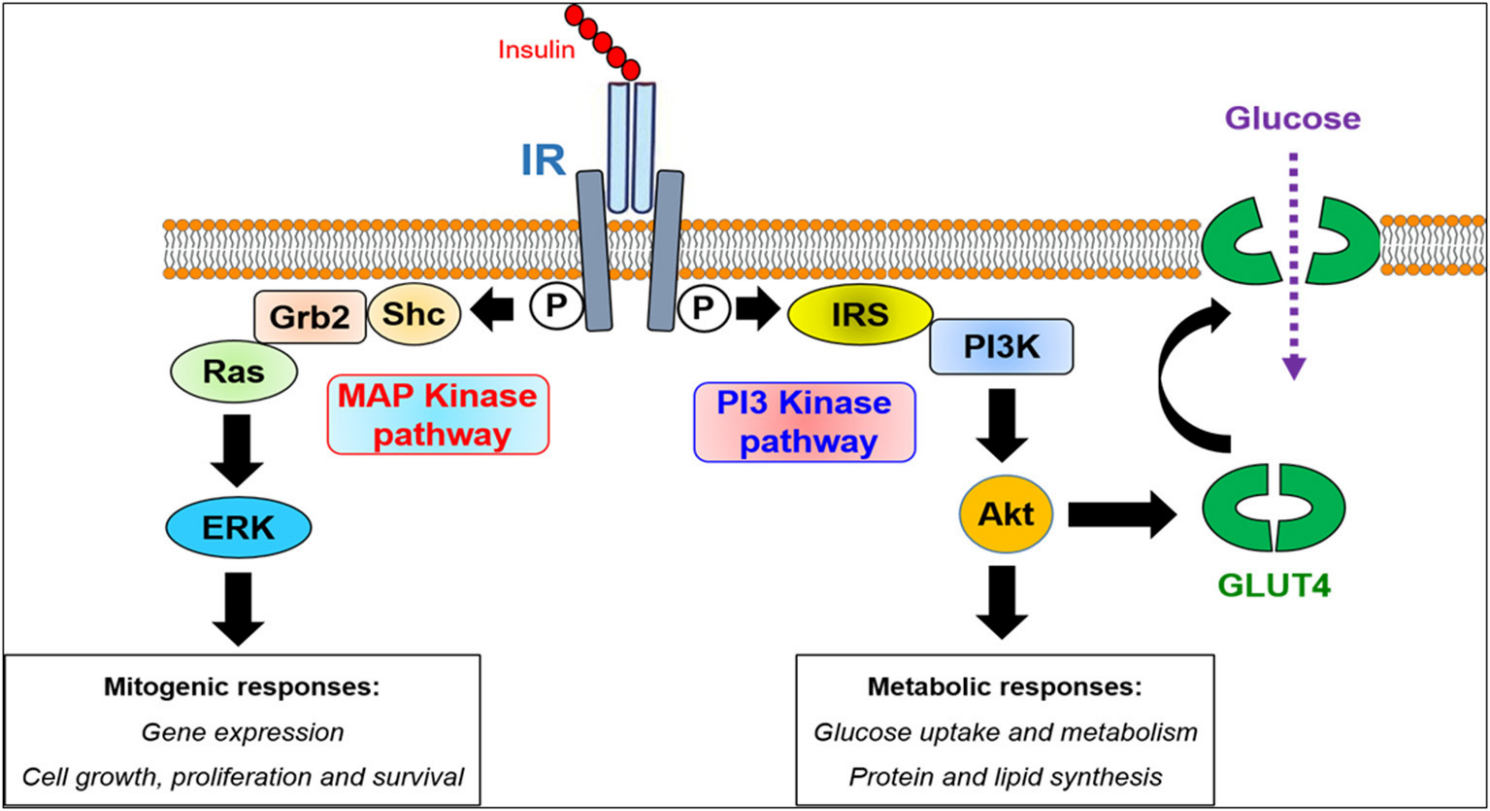
Intracellular insulin receptor signaling. The binding of insulin to its cell surface receptor (IR) in the major target tissues results in receptor activation associated with its phosphorylation (P) at tyrosine residues in the cytoplasmic domain of IR. This constitutes the signal for the recruitment of various cytoplasmic scaffolding and signaling proteins plausibly controlling two major downstream signaling pathways, the PI3-kinase and the MAP kinase pathways. The PI3-kinase pathway mediates the metabolic responses of insulin through the activation of the canonical kinase AKT controlling the expression and the translocation of the glucose transporter GLUT4 thereby glucose uptake as well as the metabolism of proteins and lipids. However, the MAP kinase pathway triggers the mitogenic responses of insulin *via* the activation of the canonical Ras/Raf/ERK1/2 cascade controlling the expression of genes involved in cell proliferation and growth.

To investigate the putative pharmacological targeting of IR by CM *in vitro*, the human embryonic kidney (HEK293) and the hepatocarcinoma (HepG2) cells were used. Indeed, HEK293 cells were used for the transient expression of IR using the mammalian expression plasmid, while HepG2 cells were used for the study on the endogenously and native expressed receptor. These cell lines were used to assess the activation of the receptor at the plasma membrane and its downstream signaling pathways leading to glucose uptake by the cells upon treatment of cells, either with insulin used as a positive control or with the different CM protein fractions. At the molecular level, insulin binds and activates its receptor (IR) belonging to the receptor tyrosine kinase family promoting the autophosphorylation of the intracellular domains of the receptor at tyrosine residues leading to the recruitment of IR substrate (IRS) proteins (Figure 4) (59). This constitutes the key step in IR signaling, since IRS proteins will be in turn phosphorylated by IR on tyrosine residues allowing the recruitment of many other intracellular signaling proteins including the phosphoinositide 3-kinase (PI3-kinase), the growth factor receptor bound protein 2 (Grb2), and the SH2 containing protein tyrosine phosphatase-2 (SHP2) (59). Depending on the signaling pathway engaged, insulin can stimulate either the metabolic or the mitogenic/growth pathways (Figure 4). The metabolic pathway is mostly controlled by the IRS/PI3-kinase/protein kinase B (AKT) axis and involved in the regulation of glucose, lipid and protein metabolism (59). Indeed, the IRS/PI3-kinase/AKT pathway is critical for the expression as well as the translocation of the glucose transporter 4 (GLUT4), from the intracellular vesicles to the cell surface (Figure 4). The mitogenic pathway is characterized by the activation of the mitogen-activated kinase (MAP Kinase) pathway *via* Grb2/SOS/Ras cascade leading to the phosphorylation of the extracellular signal-regulated kinases 1/2 (ERK1/2) (59) (Figure 4). This pathway, however, controls cell proliferation and apoptosis. Accordingly, the phosphorylation of the two canonical kinase pathways and proteins, AKT and ERK1/2, constitutes the read out for the activation of the downstream signaling pathways of IR (59).

**Figure 5:**
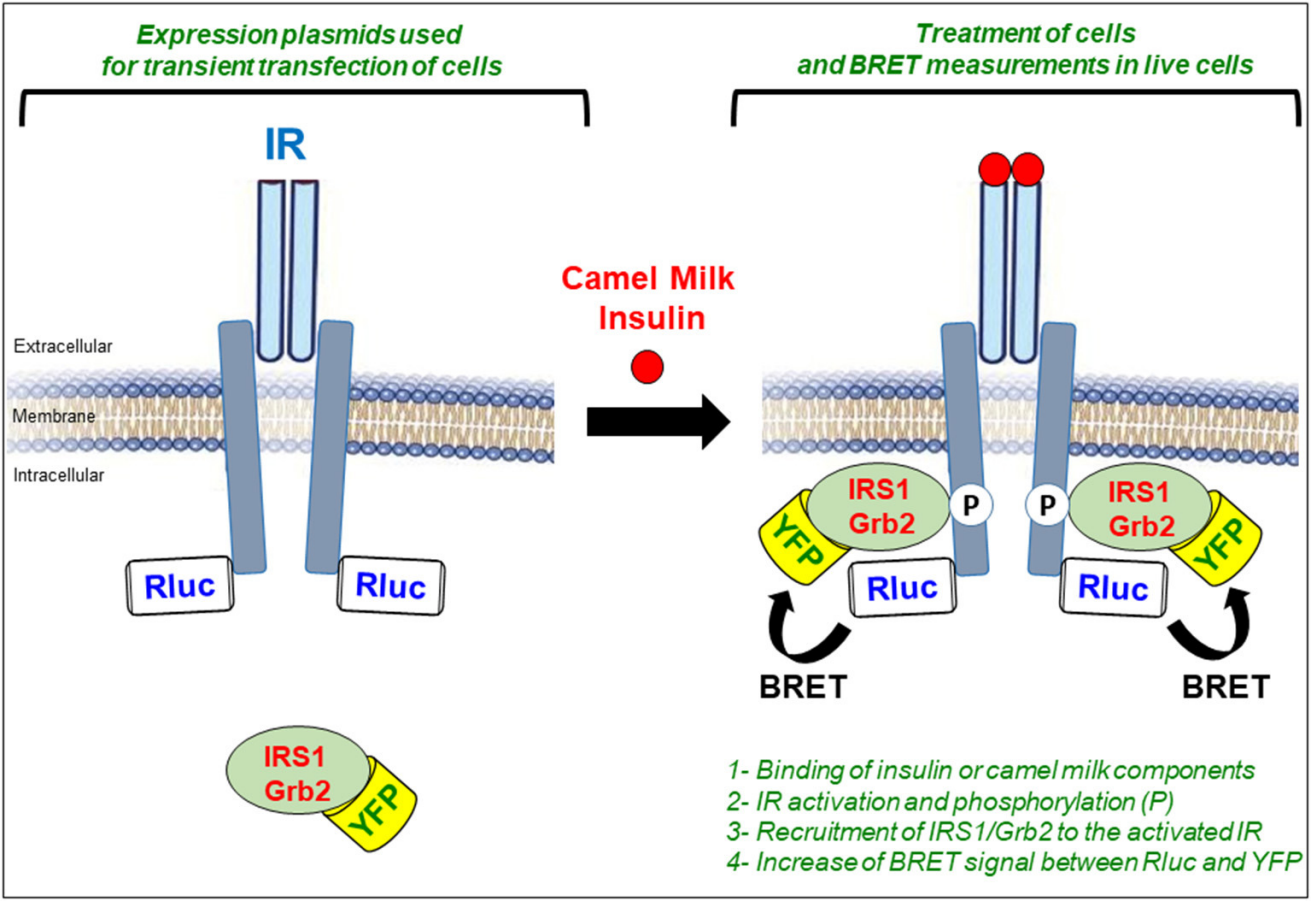
Representative figure highlighting the principle of BRET assay to assess IR activation. BRET is a proximity-based assay carried out in live HEK293 cells transiently co-transfected with plasmids encoding for Rluc-tagged IR (BRET donor) and YFP-tagged IRS1/Grb2 (BRET acceptor). Cells are then treated either with insulin as a positive control or with CM fractions in time and dose-dependent manner and BRET signals are measured in live cells. Activation of IR results in the translocation of IRS1/GRb2 to IR reducing their physical proximity. This is translated in the increase of the BRET efficiency between the BRET donor (Rluc) and the BRET acceptor (YFP) sensors.

For the IR activation assay, we used the previously described biophysical bioluminescence resonance energy transfer (BRET) technology to assess the activation of the receptor in real-time and live cells (32, 33, 51, 60). BRET is a protein proximity-based assay that in our case measures the physical translocation of the cytosolic proteins, IRS1 and Grb2, to IR upon its activation and phosphorylation by insulin or CM fractions (Figure 5). For this, the proteins are first genetically modified at the level of their cDNA integrated in the appropriate mammalian expression plasmids for cell transfection. Indeed, IR is C-terminally fused with the *Renilla* luciferase enzyme (Rluc) used as BRET donor and IRS1 or Grb2 are fused to the yellow fluorescent protein (YFP or its variant Venus) used as BRET acceptor (Figure 5). Their respective plasmids are then transiently transfected in HEK293 cells and BRET signals are measured in 96-well format directly on live cells treated or not either with insulin as a positive control or with the different CM fractions. Thus, the activation of IR in the cells can be detected by the increase in the BRET signals between IR-Rluc and IRS1/Grb2-YFP resulting from the reduction in the physical proximity between the proteins (Figure 5). In parallel with the BRET assay, the phosphorylation status of IR and its two canonical kinases, AKT and ERK1/2, are also investigated in both HEK293 and HepG2 cells using SDS-PAGE followed by Western blot analysis. Finally, glucose uptake in HepG2 cells was also examined to link our BRET and phosphorylation data to an integrated cellular response relevant to diabetes and glucose homeostasis (32, 33, 51).

## CM Proteins Pharmacologically Target IR

The seminal study by Abdulrahman et *al*. reported for the first time the evidence for the pharmacological modulation of IR activity by CM proteins in HEK293 cells (51). This was demonstrated primarily using BRET technology. Indeed, CM obtained from breeds in Saudi Arabia was fractionated into two major fractions, caseins and whey proteins, constituting the major proteins of the mammalian milk. The study showed that neither caseins nor whey proteins (1 mg/ml) had any effect on the activity of IR when applied individually (51). This may be used as a solid argument to rule out the possible implication of CM insulin due to its low levels in 1 mg/ml of the total whey proteins. However, the combination of CM whey proteins but not the casein fraction with insulin led to a potentiation of insulin-Induced BRET signals suggesting a positive allosteric action of CM whey proteins on insulin and its receptor (51). Moreover, different CM whey protein fractions were obtained by differential gel centrifugations and chromatography showing that proteins ranging from 10 kDa to 50 kDa activated IR (51). These fractions were also positive on IR activity suggesting that the bioactive protein may exist within these molecular weight ranges. Intracellularly, the study also reported that CM proteins differentially activated the downstream kinases by promoting the phosphorylation of ERK1/2 but not significantly AKT in HEK293 cells (51). Overall, the study demonstrated for the first time the bioactivity of CM proteins at the molecular level and using a cellular system and revealed the potential positive allosteric modulation of IR by CM proteins. This later opened very exciting follow-up studies on CM and its pharmacological targeting of IR as a plausible molecular and cellular basis for the antidiabetic properties of CM (11).

## Identification of IR Activating Bioactive Peptides From CM Whey

Later, we investigated more in depth the bioactivity of CM proteins and derived peptides on IR activity and its downstream signaling pathways as well as glucose uptake response (33). This was carried out using CM from breeds in the United Arab Emirates with the focus on CM whey proteins based on our previous study in Saudi Arabia (51). As stated above, this was based on well-known gastric digestion and processing of CM upon its consumption by gastric proteases and endopeptidases releasing peptide and/or amino acid molecules in the gut. Such peptides/amino acids are thereafter transported through the intestine into the circulation and exert their bioactivity at the level of different target tissues. Therefore, we used the approach of CM fractionation by hydrolysis of the raw whey proteins with the key gastric endopeptidases, including pepsin, to generate various hydrolysates and peptide fractions (33). The hydrolysis by pepsin for 2–6 hours led to generation of various hydrolysates that were characterized by reverse-phase ultra-performance liquid chromatography followed by their sequencing for peptide content. The analysis showed heterogeneous peptide content in each CM whey hydrolysate and the different peptide fractions were first profiled for their inhibitory effect on the enzyme DPP-4 *in vitro*. From this profiling, four hydrolysates named, H3, H5, H11, and H12, were selected with differential action and potency (given in IC_50_) on DPP-4 as shown in Table 1 (33). Then, the selected CM whey protein hydrolysates were functionally examined for their activity on IR in both HEK293 and HepG2 cells using both BRET and phosphorylation assays (33). This study first confirmed the bioactivity of CM whey proteins and their derived hydrolysates on IR in a cellular system with different extent and percentage of action compared to insulin (Table 1). By contrast to our previous observations in Saudi Arabia (51), we observed that CM whey proteins activated IR in a dose-dependent manner by increasing the BRET signals between IR-Rluc and IRS1-YFP and by promoting the phosphorylation of the receptor as well as its canonical downstream kinases, AKT and ERK1/2. This was observed in the transfected HEK293 cells and the native HepG2 cells endogenously expressing IR (33). However, such an observation suggests the existence as well as the implication of CM insulin in CM whey fraction. Whether the differences are due to the breeds and their environment (UAE *vs*. Saudi Arabia) or simply to the experimental or technical reasons, this constitutes an interesting aspect that requires further investigations. In addition to their inhibitory action on DPP-4, the four CM whey-derived hydrolysates (H3, H5, H11, and H12) significantly, and differentially, activated IR in a dose-dependent manner in HEK293 and HepG2 cells (Table 1) (33). Similarly to the raw CM whey proteins, the effects was associated with an increase of the BRET signals between IR-Rluc and IRS1-YFP and the phosphorylation of the receptor as well as the kinases AKT and ERK1/2. The maximal response was reached with 1 mg/ml of the hydrolysates. Moreover, the CM whey proteins and their derived hydrolysates promoted glucose uptake in HepG2 cells linking such a crucial integrated cell response with IR activation and its downstream signaling pathways (33). Together, these findings further confirm the pharmacological targeting of IR by CM proteins and peptides. At the molecular and cellular levels, this may imply either a direct or an indirect action of CM whey proteins and their derived hydrolysates on IR at the surface of the cells.

**Table 1:** Biochemical and pharmacological characteristics of CM hydrolysates and LF purified from CM on DPP-4 inhibition and IR activation and modulation.

**CM hydrolysates**	**Peptide content**	**IC_**50**_ (mg/ml) of DPP-4 inhibition**	**IR activation (%) individual treatment**	**IR activation (%) combined treatment**	**Log EC_**50**_ of insulin-induced IR activation upon co-treatment**
Insulin	ND	ND	100^a^	100^a^	−6.28 ± 0.07^a^
Whey proteins	ND	8.46 ± 0.053^a^ (*n* = 3)	80 ± 8^b^ (*n* = 8)	184 ± 18^b^ (*n* = 8)	−7.13 ± 0.14^b^ (*n* = 5)
H3	CCGM PAGNFLM NGLMHR MFE	0.44 ± 0.014^b^ (*n* = 3)	67 ± 13^c^ (*n* = 5)	183 ± 19^b^ (*n* = 5)	−7.29 ± 0.34^b^ (*n* = 5)
H5	FM PAVACCL PPLPCHM YDFL LP	8.26 ± 0.424^a^ (*n* = 3)	51 ± 8^d^ (*n* = 8)	162 ± 17^b^ (*n* = 8)	−7.63 ± 0.35^b^ (*n* = 5)
H11	LRPFL LRFPL LC HSGF	0.74 ± 0.035^b^ (*n* = 3)	76 ± 16^b^ (*n* = 5)	188 ± 23^b^ (*n* = 5)	−7.50 ± 0.34^b^ (*n* = 5)
H12	MM PAGNFLP PVAAAPVM FCCLGPVPP MLPLML PFTMGY	2.24 ± 0.003^c^ (*n* = 3)	70 ± 9^b^ (*n* = 8)	157 ± 19^b^ (*n* = 8)	−7.46 ± 0.39^b^ (*n* = 5)
LF	ND	ND	112 ± 20^a^ (*n* = 4)	234 ± 11^c^ (*n* = 5)	−6.71 ± 0.25^a^ (*n* = 5)

*The values are Mean ± SD (for IC_50_) or Mean ± SEM (for % and EC_50_ of IR activation). Within the same column, values with different superscript letters are significantly different (p < 0.05). IC_50_, concentration inducing 50% of DPP-IV inhibition; EC_50_, concentration inducing 50% of IR activation. [Adapted from (32) and (33)]*.

**Figure 6:**
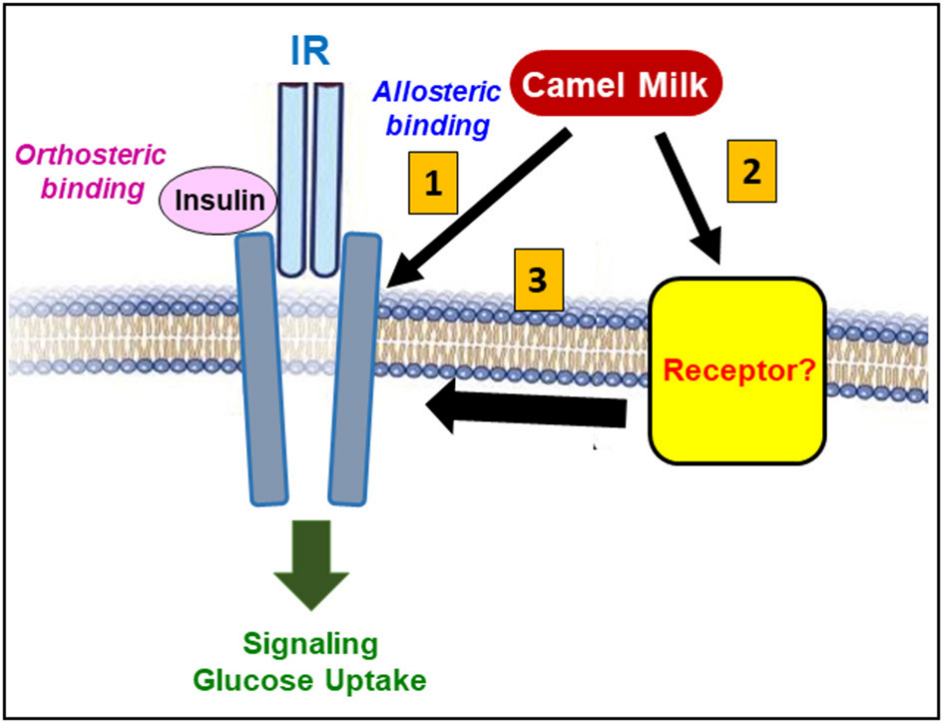
Putative model highlighting IR targeting by CM. Overall, our recent studies on the effect of CM on IR demonstrates the positive action of CM proteins/peptides on IR activity and its downstream signaling pathways. At the molecular level, our data exclude the direct binding of CM proteins/peptides on the insulin's binding site (orthosteric site) in IR. However, CM might plausibly activate IR either through an allosteric binding site leading to the positive modulation of IR and its related signaling pathways (1), or *via* a binding to another unknown receptor (2) that may synergistically/allosterically crosstalk or transactivates IR (3).

The use of the competitive IR antagonist peptide, S961, that blocks insulin binding and effect, revealed very interesting points regarding the putative mode of action of CM fractions on IR. Indeed, S961 fully blocked insulin-promoted response including the increase of BRET between IR-Rluc and IRS1-YFP and IR phosphorylation (33). However, S961 did not block the positive effect of CM whey proteins and their derived hydrolysates on IR activation when individually applied (33). Such an observation is somehow intriguing since the different responses mediated by CM fractions on IR activity and its downstream signaling pathways were similar and consistent with insulin's effect. Thus, the blockade by S961 of insulin binding site (orthosteric binding site) in IR did not affect the positive action of CM proteins/peptides suggesting different mode of action of CM fractions potentially *via* a different binding site or the implication of a different receptor. Our findings are more consistent with either the existence of an additional allosteric binding site in IR or simply the binding of CM proteins/peptides to another unknown receptor that may crosstalk and trans-activate IR leading to its positive modulation and activation (Figure 6). This was supported by our data with the combined treatment as described below. Moreover, the ineffectiveness of IR antagonist indicates that the positive action of CM whey proteins cannot be obviously attributed to insulin as suggested above. Moreover, the bioactive CM hydrolysates cannot be derived from insulin since a recent study reported that the digestive enzymes completely degraded CM insulin as well as its activity detected by ELISA assay (24). The authors suggested that insulin may not be involved in the antidiabetic action of CM as it would be fully degraded by the digestive tract proteases and thereby it may not reach the blood circulation as an intact hormone (24).

## Synergistic/Positive Allosteric Modulation of IR by CM Whey Proteins/Peptides

The other interesting aspect of the pharmacological effect of CM on IR was revealed by the combined treatments of cells with insulin and either the raw CM whey proteins or their derived hydrolysates. Indeed, the combination resulted in a potentiation of insulin-mediated IR activation associated with an increase of insulin potency and efficacy observed in both BRET and phosphorylation assays compared to insulin (Table 1) (33). This suggests the existence of synergistic and/or positive allosteric interaction between CM fractions and insulin at the molecular level of the IR protein. This finding was consistent with our previous study on the raw CM whey proteins from breeds in Saudi Arabia indicating that such a synergistic/allosteric action may be a general feature of CM on IR function (51). In addition, at the physiological level, this observation may explain the previous *in vivo* studies reporting that CM consumption reduced the insulin doses required for glycaemia correction in diabetic rat models (22, 50). From the pharmacological point of view, such a synergistic and/or positive allosteric action suggests that CM proteins/peptides may directly or indirectly induce and/or stabilize IR in a most favorable active conformation leading to further IR activation and signaling (Figure 6). Accordingly, as stated above we speculated about the possible existence of an additional allosteric binding site in IR through which CM proteins/peptides bind and exert their pharmacological effect on IR. Alternatively, CM proteins/peptides may target and engage another receptor that would allosterically interact with IR and positively modulate its activity (Figure 6). Both possibilities are supported by the data with the competitive IR antagonist peptide (S961) which binds to the orthosteric site in IR and abolished the potentiating effect of CM whey proteins and their derived hydrolysates on insulin-mediated IR activation (33). This demonstrates that the binding of insulin in its orthosteric site on IR and the active conformation of IR induced by insulin both are required for the synergistic and/or positive allosteric action of CM proteins/peptides. The existence of an allosteric binding site in IR was previously reported using selective IR antibodies shown to positively modulate insulin-mediated IR activation and signaling (61, 62). With this line, the positive allosteric modulation of IR by CM proteins/peptides may be a valid scientific rationale to explain the antidiabetic properties of CM. Moreover, this opens therapeutic perspectives for the potential applications of CM-derived proteins/peptides especially in type-2 diabetes and insulin resistance situations where insulin's action needs to be corrected and improved.

## CM Lactoferrin As a Candidate for IR Activating Bioactive Protein

The main question and aim remain the identification of the antidiabetic agent(s) contained in CM. Some studies suggested that insulin and/or insulin-like peptides could be the antidiabetic agents of CM (50), but this still needs to be further evidenced and experimentally demonstrated. As stated above, our recent studies demonstrated the targeting of IR by CM whey proteins at the cellular and molecular levels. In addition, the fractionation and hydrolysis of CM revealed bioactive peptide fractions activating IR and synergistically/allosterically modulate its activity. This constitutes an important one-step further advance toward the identification of the antidiabetic CM molecules. Sequencing of CM hydrolysates may guide us to identify the exact bioactive peptide(s) (33). However, our results do not indicate the exact CM proteins from which these hydrolysates were generated. Moreover, none can exclude the implication of intact CM proteins in the antidiabetic effects in the whole organism. Among these proteins, lactoferrin (LF) is of special interest since it represents an interesting protein in milk whey due its various bioactivities and variable content (5, 25, 53), ranging from about 1% to 7% depending on the studies, the breeds, the type of milk (human, cow, camel), and the lactation stage (52, 53, 58, 63, 64). For instance, LF concentrations in camel and cow milk vary from 0.2 mg/ml to 5 mg/ml with higher levels in camel milk than in cow milk (52, 53, 58, 63, 64). From a functional perspective, LF is known to have several beneficial biological activities including, antiviral, antibacterial, antiproliferative, and antidiabetic (42–44, 55–58, 65). In addition, LF was used for different dietetic and clinical purposes showing functional effects as an intact protein and ultimately its resistance to gastric digestion (58).

In the context of diabetes, LF has been reported to have implication in diabetes and its associated disorders such as insulin resistance, inflammation, and obesity (42–44, 55–57). For instance, in diabetic patients the circulating LF was found negatively associated with hyperglycemia while it was positively correlated with insulin sensitivity and anti-inflammatory responses (44). In addition, studies showed that LF significantly improved insulin sensitivity in type-2 diabetic patients (6). Finally, LF considerably improved the levels of key metabolic parameters (HbA1c, body-mass index and lipids) of type-2 diabetic infants (42). At the molecular level, LF increased insulin-mediated AKT phosphorylation in hepatocytes and adipocytes suggesting an effect on IR (43) with a potential implication in glucose transport where AKT plays a pivotal role (59).

In this context, we hypothesized that LF might be one of the antidiabetic proteins of CM. For this, we successfully purified LF form CM as previously reported (65, 66) and profiled its putative bioactivity on IR function in cells (32). We obtained similar data as with the whole CM whey proteins or their hydrolysates (33, 51). Indeed, the purified LF activated IR in a dose-dependent manner by increasing BRET signals between IR-Rluc and IRS1-YFP and promoting its phosphorylation in HEK293 and HepG2 cells (Table 1) (32). Moreover, LF increased the phosphorylation of AKT and ERK1/2 in both cell lines (32). Like with CM whey proteins and hydrolysates, LF, at a saturating dose of 1 mg/ml, had a synergistic effect on IR when it was combined with insulin (Table 1). Blockade of IR by its competitive antagonist (S961) had no effect on LF-mediated IR activation, whereas it completely blocked its synergistic effect (32). Interestingly, LF purified from cow milk also activated IR and its downstream signaling pathways in HEK293 and HepG2 cells (32). Overall, our data further demonstrate the functional activity of milk LF (cow and camel) at the cellular and molecular levels. Moreover, the pharmacological targeting of IR by LF from CM constitutes a further solid molecular basis for the beneficial effects of CM in diabetes with potential applications of the intact LF as an antidiabetic agent.

## Concluding Remarks

Undoubtedly, the antidiabetic properties are the most studied and investigated aspects of CM, as nutritional and medicinal diet, using *in vitro* and *in vivo* models. Our recent studies and findings can be considered seminal in this field providing for the first time a molecular and cellular basis that could explain the antidiabetic effects of CM (Figure 6). Indeed, the solid evidence for the pharmacological targeting of insulin function and its receptor (IR) at the cellular and molecular levels constitutes the scientific rationale of such beneficial effects of CM. Moreover, our fractional and analytical approach using a cellular system has revealed the existence of bioactive components in CM targeting IR, either as intact proteins (i.e., lactoferrin) or peptides (CM whey hydrolysates). Our next aim is to synthesize the positive CM bioactive peptides and to validate them for their functional effects on IR and glucose uptake. This opens promising perspectives for the identification of the bioactive CM agent as well as the development of CM-based therapeutic interventions with potential antidiabetic applications. Of course, our *in vitro* finding needs to be confirmed and further characterized in an *in vivo* model, and we believe that our work will definitely inspire different research groups investigating the antidiabetic properties of CM. The identification of the exact antidiabetic agent(s) in CM still represents a crucial aspect, which indeed requires further efforts and appropriate tools and approaches. Based on our studies, the synthetic peptides generated and identified from CM whey hydrolysates and LF purified from milk may be good candidates to be tested in *in vivo* systems, either individually or in combination with insulin, for their putative effects on insulin-dependent responses. As more and more are gleaned about these intricacies, this would be certainly instrumental in the development of CM-based therapeutics interventions in near future. We hope that this review along with our previous research studies will pave the way for more research groups to investigate the effect of camel milk on insulin receptor and more generally on the molecular mechanisms of the antidiabetic properties of camel milk.

## Author Contributions

IA wrote and designed the structure of the manuscript and made the figures. FK wrote the part of the manuscript related to lactoferrin. SM contributed to the introduction and conclusion parts of the manuscript. MA wrote and designed the structure of the manuscript and made the figures. All authors contributed to the article and approved the submitted version.

## Funding

This work was supported by a Center-based grant from Zayed Center for Health Sciences, the United Arab Emirates University (UAEU-ZCHS) (Grant Number #31R235) and the UAEU postdoc grant (Grant Number #31R241).

## Conflict of Interest

The authors declare that the research was conducted in the absence of any commercial or financial relationships that could be construed as a potential conflict of interest.

## Publisher's Note

All claims expressed in this article are solely those of the authors and do not necessarily represent those of their affiliated organizations, or those of the publisher, the editors and the reviewers. Any product that may be evaluated in this article, or claim that may be made by its manufacturer, is not guaranteed or endorsed by the publisher.
